# Habitat Association and Seasonality in a Mosaic and Bimodal Hybrid Zone between *Chorthippus brunneus* and *C. jacobsi* (Orthoptera: Acrididae)

**DOI:** 10.1371/journal.pone.0037684

**Published:** 2012-06-04

**Authors:** Richard I. Bailey, Clara I. Saldamando-Benjumea, Haruki Tatsuta, Roger K. Butlin

**Affiliations:** 1 Centre for Ecological and Evolutionary Synthesis, Department of Biology, University of Oslo, Oslo, Norway; 2 Facultad de Ciencias, Universidad Nacional de Colombia, Medellin, Colombia; 3 Department of Ecology and Environmental Science, Graduate School of Agriculture, University of the Ryukyus, Nishihara, Okinawa, Japan; 4 Department of Animal and Plant Sciences, University of Sheffield, Western Bank, Sheffield, United Kingdom; Field Museum of Natural History, United States of America

## Abstract

Understanding why some hybrid zones are bimodal and others unimodal can aid in identifying barriers to gene exchange following secondary contact. The hybrid zone between the grasshoppers *Chorthippus brunneus* and *C. jacobsi* contains a mix of allopatric parental populations and inter-mingled bimodal and unimodal sympatric populations, and provides an ideal system to examine the roles of local selection and gene flow between populations in maintaining bimodality. However, it is first necessary to confirm, over a larger spatial scale, previously identified associations between population composition and season and habitat. Here we use cline-fitting of one morphological and one song trait along two valley transects, and intervening mountains, to confirm previously identified habitat associations (mountain versus valley) and seasonal changes in population composition. As expected from previous findings of studies on a smaller spatial scale, *C. jacobsi* dominated mountain habitats and mixed populations dominated valleys, and *C. brunneus* became more prevalent in August. Controlling for habitat and incorporating into the analysis seasonal changes in cline parameters and the standard errors of parental trait values revealed wider clines than previous studies (best estimates of 6.4 to 24.5 km in our study versus 2.8 to 4.7 km in previous studies) and increased percentage of trait variance explained (52.7% and 61.5% for transects 1 and 2 respectively, versus 17.6%). Revealing such strong and consistent patterns within a complex hybrid zone will allow more focused examination of the causes of variation in bimodality in mixed populations, in particular the roles of local selection versus habitat heterogeneity and gene flow between differentiated populations.

## Introduction

Taxon pairs often remain in contact and exchange genes during some or all of the speciation process, and the maintenance of a bimodal distribution of genotypes or phenotypes in the face of this gene exchange is a key step towards speciation. Their geographic ranges may be strongly overlapping (sympatric) or they may form narrow parapatric hybrid zones where their ranges meet [1], [2], [3]. Understanding what causes sympatric populations and hybrid zones to be bimodal rather than unimodal helps in understanding the transition from a single species to pairs of species that can remain distinct when in contact [4].

Many hybrid zones contain unimodal distributions of genotypes or phenotypes within populations at their centre [4]. Examples include tension zones, where the primary selection pressure is reduced fitness of hybrids, such as *Podisima pedestris* grasshopper races [5], *Chorthippus parallelus parallelus* and *C. p. erythropus* grasshoppers [6] and *Heliconius erato* butterfly races [7]. They also include cases where the interacting populations are adapted to different environments and meet at sharp ecotones [8], as with the hybrid zones between *Bombina bombina* and *B. variegata* toads in Poland [9], [10], [11]. Such hybrid zones are thought to be formed and maintained by a balance between dispersal of parental genotypes into the zone and selection against hybrids, or between dispersal and recombination for quantitative traits [12]. The resulting cline has a smooth sigmoid shape with a width that is determined by a balance between these forces (w ∝ σ/√s, where w = cline width, σ = lifetime dispersal and s = selection) [1], [12].

Hybrid zones can deviate from this pattern in a number of different ways, and these deviations can be caused by a variety of different factors. While some linkage disequilibrium is always produced by dispersal into the centre of the zone [13] other forces can add to this, including genetic drift, epistasis [13], assortative mating [4], a complex initial pattern of contact [14], [15], phenological differences [16] and habitat association due to local adaptation [16], [17] or habitat preferences [18]. If strong enough, these forces can lead to the production of hybrid zones that consist of mosaics of local populations with distinct phenotypes or genotypes (mosaic hybrid zones), populations with bimodal phenotypic or genotypic distributions (bimodal hybrid zones), or a mixture of the two [16], [17]. Several such hybrid zones have been described, including *Gryllus pennsylvanicus* and *G. firmus* crickets [16], [17], [19], *Allonemobius socius* and *A. fasciatus* crickets [20], [21], *Bombina bombina* and *B. variegata* toads in Croatia [18] and in Romania [22], *Triturus cristatus* and *T. marmoratus* newts in France [23] and *Triturus vulgaris and T. montandoni* in the Carpathian mountains [14]. This variety of hybrid zone structures increases the utility of hybrid zones by allowing examination of the causes of variation in the maintenance of distinct genotypes during periods of contact.

Which of the above-listed factors are most likely to promote and maintain bimodality? In general, hybridization leads to the break-up of co-adapted gene complexes through recombination, and selection pressure towards bimodality must be maintained in spite of this in order to prevent its breakdown. Therefore, forces that require multiple traits (and hence multiple sets of genes) to stay in linkage disequilibrium in order to maintain selection towards bimodality are inherently less likely to be key factors than those that do not. For example, genes affecting male sexual signals, female preferences and reduced fitness in hybrids must covary for assortative mating to maintain bimodality. Habitat associations (combined with limited dispersal) and phenological differences also directly cause prezygotic isolation. If habitat associations are caused solely by habitat preferences, and phenological differences are merely an artefact of divergence in allopatry and not maintained by ecological selection in the contact zone, then these forces suffer the same problem as assortative mating – covariance with reduced hybrid fitness must be maintained in the face of recombination. However, if spatial or temporal variation in ecological selection maintains habitat and phenological differences respectively, then the same genes involved in ecological selection also directly cause prezygotic isolation, negating the homogenizing effect of recombination. Hence, habitat and phenological differences can play a key role in the speciation process.

*Chorthippus brunneus* (Thunberg) and *C. jacobsi* (Harz) are Gomphocerine grasshopper species in the biguttulus species complex [24], [25]. Their ranges meet in northern Spain where they form a mosaic hybrid zone in which some but not all populations with intermediate mean phenotypes have bimodal phenotype distributions [26]–[29]. Grasshoppers that belong to the biguttulus group are similar morphologically but remarkably different in male calling song [24], [25] suggesting rapid evolution of sexual signalling traits [29]. Hybrids between *C. brunneus* and *C. jacobsi* are fertile and show no detectable reduction in viability under laboratory conditions [30], but hybrid male song is selected against via female choice [31]. Studies have focused on key quantitative traits that can distinguish the two species: the morphological trait stridulatory peg number [27], [28], and either a composite score of male calling song [29] or echeme length [32].

**Figure 1:**
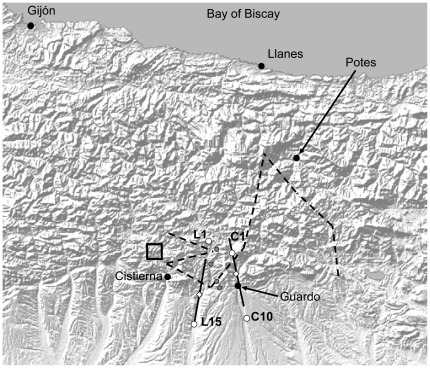
Sampling locations in Spain. Map of northern Spain indicating cline centres (open diamonds) and widths (unbroken lines) along transects 1 (the LE232, to the west) and 2 (the C615 to the east) in season 3 (the season most closely matching collecting periods by Bridle and colleagues). Collecting sites at the northern and southern ends of each transect are indicated by open circles and the four mountain sites (M1 to M4 moving from north to south) by grey-filled circles. Black circles indicate major towns and cities close to the study area. The existing fitted two-dimensional cline centre for stridulatory peg number [28] is indicated by a dashed line, and the square indicates the study area in which strong seasonality and habitat associations were previously identified [27].

There are two competing hypotheses to explain the structure of this hybrid zone. Having found little evidence of habitat effects in geographically broad combined with within-patch studies [28], [29], [33], Bridle and colleagues suggested that departures from a smooth clinal structure (mosaicism and bimodality) could be the product of long distance dispersal of parental phenotypes from outside the zone to found new populations within the zone, perhaps with preferential extinction of hybrid populations. However, in a later study [27], sampling within an area of 25 km^2^ located near the geographical centre of the zone (see Fig. 1), it was found that *C. jacobsi-*like phenotypes were predominant in mountain habitat whereas *C. brunneus-*like phenotypes were predominant in valleys, especially hay meadows and invariably in sympatry with *C. jacobsi*. In addition, in hay meadows *C. jacobsi*-like phenotypes were most frequent in July while *C. brunneus–*like individuals were more abundant in August. In this particular location, bimodality was consistently high in mixed populations.

According to later studies [27] therefore, mountain habitats contain almost solely *C. jacobsi* phenotypes, valley bottoms consistently contain sympatric mixed populations, and seasonal isolation exists but is limited to valley bottom hay meadows. Both the phenological differences and the habitat associations contribute a large proportion of the overall premating isolation, alongside relatively strong assortative mating [27]. The restriction of mixed sympatric populations to specific habitat types may indicate that isolating mechanisms (such as seasonal separation) are manifest most strongly in that habitat type, while the existence of allopatric populations nearby consistently containing one parental phenotype may indicate a role of migration between nearby populations in maintaining local bimodality [27]. However, the extent to which the study area used is representative of the hybrid zone as a whole remains unclear. Over a larger spatial scale, do hay meadows consistently harbour seasonally separated, sympatric populations and mountains *C. jacobsi*-only populations?

In this paper we ask two key questions based on the spatial and seasonal occurrence of stridulatory peg number (morphology) and echeme length (male calling song): 1. Do the seasonality and habitat associations that exist in one location persist over a larger scale? 2. Are there clinal patterns within habitat and season that are masked by collections across multiple habitat types and seasons? We also re-test, using a new sampling strategy and analysis method, whether there is a significant difference in cline width between stridulatory peg number and song, potentially indicating differences in the strength of selection operating on these traits. We use a cline-fitting procedure [12] on collections along two roughly north-south transects in valley bottom hay meadows, 25+ km in length and crossing the predicted cline centre [28], combined with collections from the intervening mountains (Fig. 1), as a means to test for seasonality and habitat associations over a larger spatial area. While the standard assumption of a balance between dispersal and selection is violated in this complex hybrid zone [29], we are nevertheless able to use cline-fitting to statistically test such important hypotheses.

## Results

### Do Seasonal Changes Match those Expected from Previous Studies?

To test for seasonal isolation, the collection was divided into blocks of time of approximately two weeks duration: collection 1 from 29^th^ June to 15^th^ July; collection 2 from 24^th^ July to 1^st^ August and collection 3 from 3^rd^ to 11^th^ August. Many but not all sites along the two transects showed the predicted pattern of a shift in mean towards *C. brunneus* values and a reduction in variance of the two traits, indicating a transition from mixed to single species, as the season progressed (Table S1).

For the cline-fitting analysis, differences in phenology between the species were expected to be revealed as (i) southward movement of the cline centre as the season progressed and hybrid zone populations became more predominantly *C. brunneus*-like, (ii) a reduction in the elevation in variance at the zone centre because in mixed sites both species were expected to be present in relatively equal proportions early in the season causing high trait variance, but *C. brunneus* were expected to predominate later on, and (iii) a reduction in cline width because *C. jacobsi*-only populations at the southern end of the zone would show no seasonal shift towards *C. brunneus* phenotypes.

**Figure 2:**
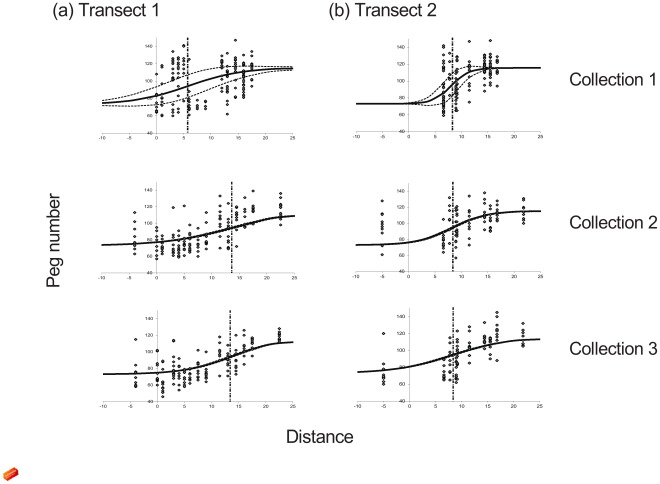
Stridulatory peg numbers and fitted clines. Stridulatory peg numbers (grey diamonds) along (a) transect 1 and (b) transect 2 for each temporally separated collection; and fitted clines (solid lines) from the best-fitting model with combined width and centre estimates for song and pegs ± fitted standard deviation (SD) in peg number (thin dashed lines). Thick vertical dashed lines indicate fitted cline centres.

Cline-fitting indicated that, for both echeme length and peg number, cline width changed twice during the season and cline centre and elevation in variance both changed between the first and second collections only (Table 1; Fig. 2). This confirms the existence of seasonal changes in population composition over a large area of the hybrid zone, and suggests a relatively abrupt seasonal change in centre and variance elevation. However, cline widths did not follow the expected pattern. Cline widths were expected to decrease as the season progressed as northern sites became increasingly dominated by *C. brunneus.* However, for transect 1 the fitted cline was widest in season 2 and narrowest in season 3 (Table 2; Fig. 2) while for transect 2 the fitted cline became wider with season. Predictions for cline centre were better supported. There was considerable southward movement of the cline centre with season in transect 1, supporting predictions, but there was only a relatively small southward shift for transect 2. For both transects and both traits, the expected large reduction in variance elevation at the zone centre later in the season was confirmed, strongly supporting the prediction of a loss of *C. jacobsi*-like phenotypes from localities that initially contained mixed populations. Addition of sites L1 and C1 at the northern end of transects in seasons 2 and 3 may have affected cline width and centre estimates because these sites were clearly mixed rather than parental *C. brunneus*-only. The transect collections therefore still failed to cross the full width of the contact zone even after addition of these sites.

**Table 1:** List of tested models for each of stridulatory peg number and echeme length, in order of AIC (best-fitting model first).

Peg number				Echeme length			
Model	Likelihood	Parameters	AIC	Model	Likelihood	Parameters	AIC
3w2c2b	−2746.97	18	5529.94	3w2c2b	−843.07	18	1722.14
2w3c2b	−2747.33	18	5530.65	3w2c3b	−841.14	20	1722.28
3w2c3b	−2746.16	20	5532.32	3w3c3b	−840.71	22	1725.42
3w3c2b	−2746.75	20	5533.5	3w3c2b	−843	20	1726
2w2c2b	−2751.13	16	5534.26	2w2c2b	−847.19	16	1726.37
2w3c3b	−2747.28	20	5534.57	2w3c2b	−845.19	18	1726.38
2w2c3b	−2750.81	18	5537.63	2w2c1b	−849.84	14	1727.68
3w3c3b	−2747.39	22	5538.79	2w1c1b	−852.73	12	1729.46
1w2c2b	−2757.75	14	5543.49	2w3c3b	−845.14	20	1730.27
2w1c2b	−2768.04	14	5564.08	2w2c3b	−847.29	18	1730.58
1w2c1b	−2774.61	12	5573.22	2w1c2b	−851.67	14	1731.33
2w2c1b	−2772.68	14	5573.35	1w2c1b	−854.11	12	1732.22
1w1c2b	−2788.5	12	5601	1w1c2b	−854.28	12	1732.56
2w1c1b	−2788.64	12	5601.28	1w2c2b	−852.54	14	1733.08
1w1c1b	−2816.17	10	5652.35	1w1c1b	−868	10	1756

w = cline width, c = cline centre relative to distances listed in Table S1, b = elevation in trait variance at the zone centre.

**Table 2:** Parameter estimates for parental means and variances for stridulatory peg number and log(echeme length) and for cline centre, width and variance elevation for each season.

Stridulatory peg number			
*C. brunneus* mean	*C. brunneus* variance	*C. jacobsi* mean	*C. jacobsi* variance
73.04	101.65	115.65	114.72
Log(Echeme length) (sec)			
*C. brunneus* mean	*C. brunneus* variance	*C. jacobsi* mean	*C. jacobsi* variance
−1.726	0.044	−0.667	0.023
Transect 1			
Centre (1, 2)	Width (1, 2, 3) (km)	*b* (1, 2) peg number	*b* (1, 2) echeme length
5.81, 13.6	19.89, 24.47, 17.76	−1763.14, −99.95	−0.899, −0.113
Transect 2			
Centre (1, 2)	Width (1, 2, 3) (km)	*b* (1, 2) peg number	*b* (1, 2) echeme length
8.02, 8.15	6.82, 13.29, 21.77	−1852.88, −81.97	−0.536, −0.092

Numbers in brackets after parameter names indicate season. Estimates are from the model with 2 centres, 3 widths and 2 elevations in variance in which cline centre and width were constrained to be identical for peg number and echeme length. Higher negative values for *b* indicate a greater elevation in trait variance in the zone centre; zero would indicate no elevation in variance. The first parameter of the quadratic (not shown) = (C. jacobsi variance – C. brunneus variance) – *b*.

### Differences in Cline Parameters between Stridulatory Peg Number and Echeme Length

There was no support for a difference in cline width or centre between echeme length and peg number, with the lowest AIC being obtained when constraining both width and centre to be a single estimate for both traits (combined widths and centres AIC = 7248.7, 26 parameters; combined centres AIC = 7250.2, 32 parameters; combined widths AIC = 7251.6, 30 parameters; sum of separate models AIC = 7252.1, 36 parameters).

### Do Mixed Populations Dominate Valley Habitat and *C. jacobsi* Dominate Mountain Habitat Over a Larger Area than Previously Described?

A mixed population is defined as any that is not dominated by parental phenotypes of a single species, and so can include both unimodal hybrid and bimodal populations. Clearly, mountain sites contained higher mean peg numbers throughout the season than expectations based on fitted clines in the valleys (Table 3), providing support for the predicted predominance of *C. jacobsi* in mountain habitat. None of the valley sites within the overlap zone contained solely phenotypes typical of one or other species (Fig. 2), confirming that valley-bottom hay meadows and crop fields consistently contain mixed populations.

**Table 3:** Comparison between mountain site mean stridulatory peg number and the mean and variance predicted from interpolation between the two valley transects.

Site	Season	Distance	Mean	Variance	Likelihood	Predicted mean	Predicted variance	Likelihood	P
M1	1	−5.32				80.24	357.74		
M1	2	−9.83	101.6	271.38	−32.52	77.76	112.06	−59.85	<0.001***
M1	3	−9.83	115.91	158.89	−32.88	78.17	112.85	−102.4	<0.001***
M2	1	−1.7				89.04	530.57		
M2	2	−7.6	118.9	186.1	−30.63	80.13	116.45	−95.52	<0.001***
M2	3	−7.6	115.5	56.28	−24.65	80.57	117.2	−78.03	<0.001***
M3	1	3.8	119.2	65.36	−13.99	105.31	443.82	−16.87	0.05<P<0.1
M3	2	−0.88	118.14	38.48	−15.78	92.36	130.12	−35.81	<0.001***
M3	3	−0.88	110.5	286.06	−32.78	92.45	130.17	−46.75	<0.001***
M4	1	5.68	119.29	41.24	−16.02	109.08	348.62	−21.89	<0.005**
M4	2	1.63	118	184.67	−30.59	97.98	131.38	−45.97	<0.001***
M4	3	1.63	115.4	87.82	−26.88	97.82	131.39	−39.16	<0.001***

Distance = distance in km directly south from the predicted cline centre.

### Does Sampling in a Single Habitat Type and Accounting for Seasonality and Variation in Parental Values Improve the Stridulatory Peg Number Cline Fit?

For stridulatory peg number, accounting for seasonality and parental variation combined with sampling in a single habitat type led to an increase in variance explained by the cline fit from a previous measure of 17.6% [28] to 61.5% (transect 1) and 52.7% (transect 2). Although the current and previous figures may not be directly comparable due to different sampling and cline-fitting procedures, the difference is large enough to indicate a substantial increase in variance explained.

## Discussion

Here we use and extend existing cline-fitting methods to (a) estimate the elevation in trait variance in the centre of the hybrid zone [34], (b) test for seasonal changes in zone width, centre and variance elevation at the zone centre, (c) allow for uncertainty in parental values of the measured quantitative traits, and (d) carry out a statistical test for differences in cline width and centre between two traits: echeme length (song) and stridulatory peg number (morphology). We find that, along two valley transects each greater than 26 km in length, plus intervening mountains, valley habitats invariably contain mixed populations and mountain habitats are dominated by *C. jacobsi*, as predicted from previous studies on a smaller spatial scale. Trait values show stronger clinal patterns within a single habitat type and season compared to previous studies, further confirming the importance of habitat effects on zone structure. Seasonal changes in population composition, with valley populations becoming more *C. brunneus*-like as the season progresses, are confirmed over a large area. Finally, we find no evidence for a difference in width – and hence no evidence for differences in selection pressures – between the purportedly neutral morphological trait stridulatory peg number and the echeme length of male calling song. While the large estimated number of genes involved in differences between *C. brunneus* and *C. jacobsi* in echeme length implies that this aspect of song may be not be under sexual selection via female choice [32], this does not constitute strong evidence for neutrality. In general, we expect selection on song in the hybrid zone, even if not within parental populations, and we would expect to detect that by measuring a reliable song trait that clearly differs between species. Hence, the lack of difference from pegs is somewhat surprising.

Width comparisons to the estimates from two-dimensional cline-fitting given by Bridle and colleagues should be treated with caution since the precise orientation of the zone is not known and, in the case of song, also because echeme length rather than a composite song score was used in this analysis.

Fitted clines correspond to some extent with previous studies [28], [29], but are much wider and centres are shifted to the south in the most closely corresponding collecting season (Fig. 1). The zigzag at the western end of the two-dimensional clines is probably due to limited sampling and is supported neither by the current study nor by similar transect collections carried out along the N621 road running north-south through Cistierna [35].

The mosaic and bimodal nature of this hybrid zone violates standard assumptions regarding the mechanisms maintaining the cline width, position and shape. If selection within the zone is strong enough relative to interbreeding and recombination to maintain bimodality, this means that trait covariance and linkage disequilibrium values at the zone centre are not caused by a balance between gene flow (introducing parental gene combinations) and recombination (breaking them up), as assumed in standard cline models for quantitative traits [12]. Therefore estimated cline width and elevation in linkage disequilibrium, trait variance and covariance at the zone centre cannot be used to secondarily estimate dispersal, the strength of selection, or the number of genes involved in selection against hybrids in this zone or others that violate standard assumptions [29]. Furthermore, the analysis assumes normally distributed trait values within each sample, which is clearly violated in bimodal populations. Nevertheless, the cline fits explain a high proportion of the among-site variation and there were clear differences between alternative models. Therefore, cline-fitting remains a valuable tool for statistical hypothesis testing in a variety of contact zones and has revealed a complex interaction between seasonal and environmental effects in this hybrid zone.

### Habitat Effects

Habitat heterogeneity clearly contributes to mosaicism and hence the maintenance of parental phenotypes in this and several other hybrid zones [16], [18], [20], [22] although there are exceptions, particularly associated with a complex pattern of initial contact [14], [15]. However, unlike other mosaic hybrid zones, in the *C. brunneus*/*C. jacobsi* hybrid zone there is no clear separation of the two parental types into distinct habitats, as one of the two habitat types consistently contains mixed populations. In a previous study [27] therefore the authors suggested that habitat heterogeneity may also be a key determinant of local bimodality, due to the influence of gene flow between populations in close proximity but with consistently differing population compositions. In unimodal hybrid zones, the taxa tend to occupy a narrower range of habitats, such as mesic valley habitats for *C. parallelus* [36]. The existence of nearby allopatric populations of one or both parental phenotypes, leading to immigration of parental gene combinations into mixed populations, may reduce the effect of recombination in breaking down the relationship between sexual signals and preferences, and hence help to maintain selection pressure towards two distinct genotypic clusters.

The fitted clines in this study coincide strongly with the transition from the Cantabrian Mountains to the flat plains to the south (Fig. 1). We hypothesize that (i) as stated above, the presence of allopatric populations of *C. jacobsi* in the mountains is a requirement for bimodality in the valleys (i.e. regional processes are important), or (ii) bimodality is maintained by within-population selection (and hence is caused by local processes and not by habitat heterogeneity) but is broken down by the weight of gene flow from *C. jacobsi*-only populations to the south. These hypotheses remain to be tested. Many bimodal hybrid zones contain local populations with only one of the two taxa present, and sympatric coexistence often involves one taxon with a broader geographic range than the other. Hence it is important to consider the involvement of regional as well as local processes in maintaining sympatric coexistence and bimodality. An example on a larger spatial scale of the importance of allopatric populations comes from the fruit fly *Rhagoletis pomonella*, in which an inversion polymorphism affecting key diapauses traits originating in Mexico has facilitated sympatric shifts to novel host plants with differing fruiting times further north in North America [37].

### Effects of Seasonal Differences in Population Composition

Seasonality has a direct influence on reproductive isolation through reducing contact between genotypes, and so would be expected to coincide with bimodality, as it does in this hybrid zone. Furthermore, valley bottom meadows may provide the best conditions for seasonal separation, for example if the conditions are more stable or favourable for grasshoppers over an extended period compared to other habitats. Such favourable conditions may allow this pre-existing isolating mechanism [24], [25] to be manifest most strongly in this habitat type. While we provide no evidence to test for reinforcement, an increase in seasonal differences between taxa caused by reinforcing selection against hybrids [38], in this habitat specifically, represents a plausible hypothesis for future testing. *Chorthippus brunneus*, which predominate later in the season in this hybrid zone, are known to begin singing in early June in central and western Europe (R. Bailey, pers. obs.) - earlier than all the collections made for this study. Mixed sites with small seasonal changes already contained large numbers of *C. brunneus* phenotypes by the start of sampling, in late June, while their numbers increased more dramatically around the beginning of August in sites with strong seasonality. This pattern warrants further examination as it may provide clues to the relationship between seasonality, bimodality and the maintenance of other isolating mechanisms such as sexual signalling systems.

### Future Directions

In order to understand how barriers to gene exchange operate during periods of contact it is important to examine the factors, both local and regional, that allow a hybrid zone or local population to be bimodal rather than unimodal. Hybrid zones such as the one between *C. brunneus* and *C. jacobsi*, with such enormous variety in the composition of local populations, can help in determining the relative importance of different contributing factors such as local selection pressures, habitat heterogeneity, seasonal separation of breeding seasons, and sexual signalling systems. This would give clues to the relative importance of different genomic, phenotypic and environmental factors during non-allopatric speciation and coexistence during secondary contact.

## Materials and Methods

### Study Site

Collections were made between June and August 2001. Transects were based around two roughly parallel roads, the LE232 to the west and C615 to the east, running approximately north-south along major valleys in Castilla y Leon, Northern Spain (Fig. 1). Transects were chosen to coincide with the fitted cline centres for stridulatory peg number and song structure from previous studies [28], [29] (Fig. 1). Fifteen sample sites (referenced L1 to L15, north to south) were chosen at approximately 1 km intervals along the LE232 (a larger gap between L1, which was only sampled in seasons 2 and 3, and L2), and ten sites (labelled C1 to C10, north to south) were chosen at 0.5 to 2 km intervals along the C615 (a larger gap between C1, only sampled in seasons 2 and 3, and C2; Fig. 1, 2). Sampling was extended in seasons 2 and 3 to include sites L1 and C1 because it was clear that transects did not cover the full width of the zone of overlap. Sample sites were adjacent to the road and were composed of hay meadows, alfalfa (*Medicago sativa*) fields and oat and wheat crops. Four further sites (M1 to M4, from most northerly to most southerly respectively; Fig. 1) were chosen to collect grasshoppers in mountain habitat; these sites were amongst oak trees (*Quercus* sp) or in small, isolated meadows or pasture and were at least 0.5 km away from the aforementioned roads. All sample sites were less than 50 m×50 m in extent. All necessary permits were obtained for the described field studies. Collection permits were provided by the Instituto para la Conservacion de la Naturaleza (ICONA) and the Parque Nacional de Covadonga, Spain.

### Sampling, Song Recording and Morphological Analysis

The collection was divided into blocks of time of approximately two weeks duration: collection 1 from 29^th^ June to 15^th^ July; collection 2 from 24^th^ July to 1^st^ August and collection 3 from 3^rd^ to 11^th^ August. In total, 964 adult male grasshoppers, identified by song as *C. brunneus, C. jacobsi* and their hybrids were collected using nets and stored in 96% ethanol.

Songs of 830 males were recorded in the field prior to capture at approximately 30 cm distance from the microphone. The recordings were made on C90 Chrome audiocassettes using a Sony professional tape recorder (WM-D6C, Sony, Tokyo, Japan) and a Senheisser ME66 unidirectional microphone (Wennebostel, Germany). No sound filtering was used at this stage. For digitization, songs were played back using the Sony professional recorder and filtered using a Fern Development EF05-03 LP/HP filter with a hi-pass at 800 Hz. Male calling songs, with an average of 5 echemes, were digitized with Cool edit 96 (Symtrilium software corporation, Phoenix, U.S.A) at a sampling rate of 44100 samples per second and.wav documents were saved for further song analysis using the “Echeme extractor” software [32]. “Echeme extractor” was used for the identification of two calling song traits: echeme length and syllable length [29]. However, for the purposes of this investigation echeme length was chosen for the identification of *C. brunneus*, *C. jacobsi* and hybrids since it produces a robust separation between parental samples [39] and it is the song trait least affected by environmental variables such as temperature, method of measurement, or recording quality [32].

Stridulatory peg number was counted for each individual sampled in the hybrid zone. One leg was removed and fixed on a transparent glass slide and pegs were counted at 50x magnification as in [28].

### Cline-fitting Procedure

Echeme length values were log-transformed to improve normality, and prior to analysis these negative values were further transformed as (echeme length +5)×10 in order to improve the efficacy of likelihood analysis by rendering them positive and increasing the variance estimates above 1. Cline-fitting was carried out using a one-dimensional approach. The two transects were close to straight lines and were expected to cross the centre of the hybrid zone between *C. brunneus* and *C. jacobsi* perpendicular to its generally east-west course (assuming that the zigzag at the western end is a sampling artefact; Fig. 1; [29]). Cumulative straight-line distances from the northernmost sample sites in seasonal collection 1 were used (see Table S1).

Under a variety of models, clines in allele frequencies or mean phenotypes approximately follow a tanh curve [12]. For a quantitative trait, this tanh curve can be described by the position of the centre point *c* and the width *w* of the cline, and the mean values for the two ‘pure’ populations. A quadratic relationship between within-population trait variance and distance was also added to the model to accommodate both (i) differences in variance between the parental species and (ii) the expected elevation in variance at the zone centre (*b*, the second parameter of the quadratic; [34]). The value of these quantitative traits is not fixed within parental populations, and parental values are also estimated with some error. For this reason the likelihood of the model was calculated from the combined likelihoods of the cline parameters (*c*, *w* and *b*) and a normally distributed likelihood surface of parental trait values, produced using means and standard errors from collections of parental populations from outside the hybrid zone.

Analyses were carried out in GenStat v. 10 (VSN International, Ltd). For each model tested, we searched for the maximum likelihood using a Metropolis algorithm [40], [41] and recycling the parameters *c*, *w*, *b*, *C. brunneus* mean and variance, and *C. jacobsi* mean and variance. The parental population values were constrained to be identical for the two transects for each run. Each model was run 6 times for 10,000 iterations; 3 times with random starting values followed by 3 more runs, in each case choosing the current best parameter estimates as starting values and narrowing the range of possible values in order to home in on the best fit.

### Seasonal Changes in Cline Parameters

We tested (i) the null hypothesis (no seasonal change) using a single estimate of *c*, *w* and *b* per transect, (ii) an abrupt seasonal change using two estimates, one for collection 1 and one for collections 2 and 3 combined (chosen based on [27]), or (iii) a gradual change using three estimates (one for each of collections 1, 2 and 3). Both transects and all seasons were fitted simultaneously, with separate estimates of *c*, *w* and *b* for each transect and (where appropriate) season. The best-fitting models for echeme length and peg number were determined using the Akaike Information Criterion (AIC; [42]).

### Differences in Cline width between Stridulatory Peg Number and Echeme Length

The same model provided the best fit for both echeme length and peg number (Table 1). Therefore to test for significant differences between echeme length and peg number in cline centre or width, analysis of the two traits was run simultaneously for this model (3*w*, 2*c*, 2*b* per transect) with likelihoods for both traits combined into a single overall likelihood calculation. This time analyses began with 20,000 iterations for the three sets of random starting values due to the large number of parameters being estimated. Cline centre, width, or both, were then constrained to be the same for the two traits in each season before running the analysis again and comparing AIC values.

### Habitat Associations

For the analysis of habitat association only peg number data were considered because few recordings were obtained from the low density populations in the mountain sites. In order to compare valley with mountain habitats, we used linear interpolation between the two fitted valley transect cline centres to estimate the latitude of the cline centre at the same longitude as each mountain site, separately for each season. The distances north to south between each mountain site and its cline centre for each season were then calculated. The cline widths and elevations in variance were averaged across the two transects, and the predicted mean and variance of peg number were then calculated using the estimated position of each mountain site along the cline in each season separately. For each season and site separately, the summed negative log-likelihood of the individual peg numbers was calculated given (a) their actual mean and variance (hence with two unconstrained parameters) and (b) their predicted mean and variance from the cline fits (no unconstrained parameters) using the LLNormal procedure in GenStat v. 10 (VSN International, Ltd.). Twice the difference in negative log-likelihood with 2 degrees of freedom (the difference in the number of parameters) was compared to Chi-square tables in order to determine whether each observed sample differed from the prediction.

### Does accounting for Seasonality and Sampling in a Single Habitat Type Improve the Stridulatory Peg Number Cline Fit?

To test for an improvement in the cline fit for stridulatory peg number compared with previous studies [28], the observed means for each sample location and season were regressed on expected means from cline fitting; R^2^ from this regression estimates the proportion of variance explained by the cline fit. A different song measure was used here than in previous studies [29] so no comparison was made.

## Supporting Information

Table S1 **Latitude and longitude (decimal degrees) of sampling sites, cumulative straight-line distances (km) from the northernmost sample sites in seasonal collection 1, and sample sizes, means and variances of measured traits for each of the three temporally separated collections along each transect.**
(XLSX)

## References

[1] Barton NH, Hewitt GM (1985). Adaptation, speciation and hybrid zones.. Annu Rev Ecol Syst.

[2] Harrison RG (1990). Hybrid zones, windows on evolutionary process.. Oxford Surveys in Evolutionary Biology.

[3] Harrison RG (1993). Hybrids and Hybrid Zones: Historical Perspective..

[4] Jiggins CD, Mallet J (2000). Bimodal hybrid zones and speciation.. Trends Ecol Evol.

[5] Hewitt GM, Nichols RA, Barton NH (1987). Homogamy in a hybrid zone in the Alpine grasshopper *Podisma pedestris*.. Heredity.

[6] Butlin RK, Ritchie MG, Hewitt GM (1991). Comparisons among morphological characters between localities in the *Chorthippus parallelus* hybrid zone (Orthoptera: Acrididae).. Philos Trans R Soc Lond B Biol Sci.

[7] Mallet JLB (1993). Speciation, Raciation, and Colour Pattern Evolution in *Heliconius* Butterflies: Evidence from Hybrid Zones..

[8] Kruuk LEB, Baird SJE, Gale KS, Barton NH (1999). A comparison of multilocus clines maintained by environmental adaptation or by selection against hybrids.. Genetics.

[9] Sanderson N, Szymura JM, Barton NH (1991). Variation in mating call across the hybrid zone between the fire-bellied toad *Bombina bombina* and *B. variegata*.. Evolution.

[10] Szymura JM, Barton NH (1986). Genetic analysis of a hybrid zone between the fire bellied toads *Bombina bombina* and *Bombina variegata*, near Cracow in southern Poland.. Evolution.

[11] Szymura JM, Barton NH (1991). The genetic structure of the hybrid zone between the fire-bellied toads *Bombina bombina* and *B. variegata*: comparisons between transects and between loci.. Evolution.

[12] Barton NH, Gale KS (1993). Genetic analysis of hybrid zones..

[13] Barton NH (1983). Multilocus clines.. Evolution.

[14] Babik W, Szymura J, Rafinski J (2003). Nuclear markers, mitochondrial DNA and male secondary sexual traits variation in a newt hybrid zone (*Trituris vulgaris* x *T. montandoni*).. Mol ecol.

[15] Lunt I, Hewitt GM (1998). mtDNA phylogeography and postglacial patterns of subdivision in the meadow grasshopper *Chorthippus parallelus.*. Heredity.

[16] Harrison RG, Rand DM (1989). Mosaic hybrid zones and the nature of species boundaries..

[17] Ross CL, Harrison RG (2002). A fine-scale spatial analysis of the mosaic hybrid zone between *Gryllus firmus* and *Gryllus pennsilvanicus*.. Evolution.

[18] MacCallum CJ, Nurnberger B, Barton NH, Szymura JM (1998). Habitat preference in the Bombina hybrid zone in Croatia.. Evolution.

[19] Harrison RG, Bogdanowicz SM (1997). Patterns of variation and linkage disequilibrium in a field cricket hybrid zone.. Evolution.

[20] Howard DJ (1986). A zone of overlap and hybridization between two ground cricket species.. Evolution.

[21] Howard DJ, Waring GL, Alana Tibbets C, Gregory PG (1993). Survival of hybrids in a mosaic hybrid zone.. Evolution.

[22] Vines TH, Köhler SC, Thiel, A, Ghira I, Sands TR (2003). The maintenance of reproductive isolation in a mosaic hybrid zone between the fire-bellied toads *Bombina bombina* and *B. variegata.*. Evolution.

[23] Arntzen JW, Wallis GP (1991). Restricted gene flow in a moving hybrid zone of the newts *Triturus cristatus* and *Triturus marmoratus* in western France.. Evolution.

[24] Ragge DR, Reynolds WJ (1988). Songs and taxonomy of the grasshoppers of the *Chorthippus biguttulus* group in the Iberian peninsular (Orhtoptera: Acrididae).. J Nat Hist.

[25] Ragge DR, Reynolds WJ (1998). The Songs of the Grasshoppers and Crickets of Western Europe..

[26] Bailey RI, Lineham ME, Thomas CD, Butlin RK (2003). Measuring dispersal and detecting departures from a random walk model in a grasshopper hybrid zone.. Ecol Entomol.

[27] Bailey RI, Thomas CD, Butlin RK (2004). Premating barriers to gene exchange and their implications in a mosaic hybrid zone between *Chorthippus brunneus* and *Chorthippus jacobsi*.. J Evol Biol.

[28] Bridle JR, Baird SJE, Butlin RK (2001). Spatial structure and habitat variation in a grasshopper hybrid zone.. Evolution.

[29] Bridle JR, Butlin RK (2002). Mating signal variation and bimodality in a mosaic zone between *Chorthippus* grasshopper species.. Evolution.

[30] Saldamando CI, Tatsuta H, Butlin RK (2005). Hybrids between *Chorthippus brunneus* and *C. jacobsi* (Orthoptera: Acrididae) do not show endogenous postzygotic isolation.. Biol J Linn Soc Lond.

[31] Bridle JR, Saldamando CI, Koning W, Butlin RK (2006). Assortative preferences and discrimination by females against hybrid male song in the grasshoppers *Chorthippus brunneus* and *Chorthippus jacobsi* (Orthoptera: Acrididae).. J Evol Biol.

[32] Saldamando CI, Miyaguchi S, Tatsuta H, Kishino H, Bridle JR (2005). Inheritance of song and stridulatory peg number divergence between *Chorthippus brunneus* and *C. jacobsi*, two naturally hybridizing grasshopper species (Orthoptera: Acrididae).. J Evol Biol.

[33] Bridle JR, Vass de Zomba J, Butlin RK (2003). Fine scale ecological and genetic structure in a grasshopper hybrid zone.. Ecol Entomol.

[34] Shuker DM, Underwood K, King TM, Butlin RK (2005). Patterns of male sterility in a grasshopper hybrid zone imply accumulation of hybrid incompatibilities without selection.. Proc Roy Soc B.

[35] Bailey RI (2001). Coexistence in a mosaic overlap zone in *Chorthippus* grasshoppers..

[36] Voisin JF (2003). Atlas des Orthoptères et des Mantides de France..

[37] Feder JL, Berlocher SH, Roethele JB, Dambroski H, Smith JJ (2003). Allopatric genetic origins for sympatric host-plant shifts and race formation in *Rhagoletis*. Proc Natl Acad Sci U S A..

[38] Servedio MR, Noor MAF (2003). The role of reinforcement in speciation: theory and data.. Ann Rev Ecol Evol Syst.

[39] Bridle JR (1998). Spatial structure in a grasshopper hybrid zone..

[40] Kirkpatrick S, Gelatt CD, Vecchi MP (1983). Optimization by simulated annealing.. Science.

[41] Tregenza T, Pritchard VL, Butlin RK (2000). The origins of premating reproductive isolation: testing hypotheses in the grasshopper *Chorthippus parallelus*.. Evolution.

[42] Johnson JB, Omland KS (2004). Model selection in ecology and evolution.. Trends Ecol Evol.

